# Editorial: Decision-making experiments under a philosophical analysis: human choice as a challenge for neuroscience

**DOI:** 10.3389/fnins.2015.00288

**Published:** 2015-08-18

**Authors:** Gabriel J. C. Mograbi

**Affiliations:** Research Group on Mind and Brain, Department of Philosophy, Federal University of Mato GrossoCuiabá, Brazil

**Keywords:** decision-making, decision neuroscience, neurophilosophy, neuroethics, neuroeconomics, free will, neural correlates of decision-making

Decision-making is a complex subject in neuroscience. In the last years, considerable advances were achieved in different fields ranging from modulatory neurotransmitters to functional imaging, from neuroeconomics to neuroethics. Our research topic envisages a critical view on the state-of-the-art of decision neuroscience by means of foudational and methodological approaches to practical and empirical science. Accordingly, we exhorted contributions that deeply analyze neuroscientific experiments in both technical and philosophical ways aiming a broader understanding of the relevance, scope and limitations of decision-making experiments. Moreover, we encouraged epistemological reflections about the necessary neural mechanisms to decision-making. This topic is constituted by the following papers:

Sip et al. (2012) addresses decision to deceive and its related social pressure. Participants in the fMRI scaner were confronted by an opponent about his/her knowledge on a display's content and were rewarded for successful deception and penalized for ineffective ventures. The results, in addition to showing, as expected, that the decision to deceive is influenced by the risk of being detected and the social confrontation represented by the detection, also reveal that participants were slower when taking an honest course of action instead of taking advantage of their privileged knowledge. Also, important results concerning functional brain areas involved in the tasks are presented.

An elegant Bayesian decision model is presented in Deneve (2012) that both infers the probability of two different choices and simultaneously estimates the reliability of the sensory information on which this choice is based. Trials in which the level of difficult is higher show early sensory inputs having a stronger impact on the decision. Accordingly, the threshold collapses such that response time is shorter, tough with lower accuracy. Easy trials, by their turn, show the opposite: an increased sensory weight and a higher threshold over time, eliciting slower, but more accurate, decisions. As the model advanced by the author considers adaptive sensory weights, it could not only extract a single estimate from the sensory input, but also evaluate the uncertainty associated with it.

Osman (2012) empirically compares Choice-based decision-making and Prediction-based learning, showing that the former leads to more accurate cue-outcome knowledge. The author interprets results as suggesting that the additional demand of cognitive resources for the processing of rewards could be an explanation of its adverse effect in the decisional process. Also, a series of philosophical considerations is forwarded to question how generalizable is evidence from neuropsychology to psychology and vice-versa. In this context, the relationship of intra-level and inter-level experiments is considered.

Nakao et al. (2012) compares and disentangle two types of empirical protocols used for study of decisional processes: experiments that assign to its participants tasks in which a unique but uncertain answer is presupposed and experiments in which no unique external cued answer could be considered correct. The former is categorized as externally oriented decision-making and the latter as internally oriented decision-making. The article also uses Multi-Kernel Density Analysis (MKDA) to contrast internally and externally guided decisions in terms of recruitment of areas, to finally compare commonalities and differences between the two types of decisions.

Heinzelmann et al. (2012) discusses the practical and moral question of inappropriate behavior considering its foundations in both philosophical normative and descriptive domains. The moral implication of empirical findings in neuroscience, economics and psychology are discussed in the light of this philosophical background aiming at an understanding of the possible mechanisms of moral inappropriate actions and the decisional process that leads to them. More importantly, the paper addresses the morally important and controversial question of interventions to promote behavior improvement.

Taking as a standpoint Stephens and Anderson's (2001) already classic article, Bourgeois-Gironde (2012) aims at considering the viability of methodological transfers from behavioral ecology to experimental economics, including human choice inasmuch as it is concerned with intertemporal preferences. The author suggests that economic theories have noticeable similarities to ecological models in their assumptions and implications.

Lucci (2013) proposes an investigation of the subjective component of time in intertemporal choice (IC). The author asserts that deviations from exponential reward discounting, as a function of time, could have as a primary factor the deviation of subjective time from the calendar metric system time. Time perception, she claims, could modulate discounting. Consequently, time perception would be a fundamental component of intertemporal choice.

In Smaldino and Richerson (2012) the authors argue that current paradigms in neuroscience are focused on decisions made among a previously established set of options, although, the very generation of options has barely been studied and still to a great extent an untapped issue. The author considers various specific factors that could influence the generation of options that would be categorizable in two broadly defined domains: psycho-biological and socio-cultural.

Volz and Gigerenzer (2012) Argues that normative strategies used to decide under risk could not be generalized to all types of decision-making processes. They stress that in most of the experimental designs, the strategies to deal with risk are assumed as implicit presuppositions even if they are not applicable. They show that criteria for generating optimal solutions in decisional processes under risk could not be the best whenever uncertainty is the difficulty the agents have to cope with.

Shadlen and Roskies (2012) defends the possibility of a reconciliation of responsibility with neurobiological mechanism by philosophically reviewing presuppositions and implications of recent empirical studies in neurobiology. Instead of the more traditional account of compatibilism based on an appeal to randomness or noise as a source of freedom, they rather recognize that randomness could possibly establish the background against which policies have to be adopted.

Finally, Mograbi (2013) summarizes and critically analyses the merits, achievements, scope and limitations of each article in this present edition and also considers future directions in some of those cases. It can be taken as an extension of this editorial and constitutes a more detailed introduction to the whole edition.

## Conflict of interest statement

The author declares that the research was conducted in the absence of any commercial or financial relationships that could be construed as a potential conflict of interest.
